# Emerging Concepts in Innate Lymphoid Cells, Memory, and Reproduction

**DOI:** 10.3389/fimmu.2022.824263

**Published:** 2022-06-14

**Authors:** Rodolfo R. Favaro, Katherine Phillips, Romane Delaunay-Danguy, Kaja Ujčič, Udo R. Markert

**Affiliations:** Placenta Lab, Department of Obstetrics, Jena University Hospital, Jena, Germany

**Keywords:** innate lymphoid cells (ILCs), Natural Killer cells, innate immune memory, trained immunity, uterus, Endometrium, pregnancy

## Abstract

Members of the innate immune system, innate lymphoid cells (ILCs), encompass five major populations (Natural Killer (NK) cells, ILC1s, ILC2s, ILC3s, and lymphoid tissue inducer cells) whose functions include defense against pathogens, surveillance of tumorigenesis, and regulation of tissue homeostasis and remodeling. ILCs are present in the uterine environment of humans and mice and are dynamically regulated during the reproductive cycle and pregnancy. These cells have been repurposed to support pregnancy promoting maternal immune tolerance and placental development. To accomplish their tasks, immune cells employ several cellular and molecular mechanisms. They have the capacity to remember a previously encountered antigen and mount a more effective response to succeeding events. Memory responses are not an exclusive feature of the adaptive immune system, but also occur in innate immune cells. Innate immune memory has already been demonstrated in monocytes/macrophages, neutrophils, dendritic cells, and ILCs. A population of decidual NK cells characterized by elevated expression of NKG2C and LILRB1 as well as a distinctive transcriptional and epigenetic profile was found to expand during subsequent pregnancies in humans. These cells secrete high amounts of interferon-γ and vascular endothelial growth factor likely favoring placentation. Similarly, uterine ILC1s in mice upregulate CXCR6 and expand in second pregnancies. These data provide evidence on the development of immunological memory of pregnancy. In this article, the characteristics, functions, and localization of ILCs are reviewed, emphasizing available data on the uterine environment. Following, the concept of innate immune memory and its mechanisms, which include epigenetic changes and metabolic rewiring, are presented. Finally, the emerging role of innate immune memory on reproduction is discussed. Advances in the comprehension of ILC functions and innate immune memory may contribute to uncovering the immunological mechanisms underlying female fertility/infertility, placental development, and distinct outcomes in second pregnancies related to higher birth weight and lower incidence of complications.

## Introduction

A complex array of immunological interactions takes place in the uterine environment that is crucial for reproductive success, contributing to tissue homeostasis and renewing, placental development, and maternal tolerance (1–3). Immune cells engage in an intense cross-talk with decidual and trophoblast cells at the maternal-fetal interface. Mediated by cell-cell interactions, cytokines, extracellular vesicles, and microRNAs (miRNAs), this communication modulates the recruitment, differentiation, and education of uterine immune cells into unique phenotypes that support pregnancy (4–7).

All innate immune cells, except basophils, are eventually present in the uterus under physiological conditions (8–12). Except for Natural Killer (NK) cells, most innate lymphoid cells (ILCs) have been only superficially investigated in the context of female reproduction and pregnancy. A better characterization of the populations, functions, and mechanisms employed by ILCs in the uterine environment is fundamental to understanding reproductive processes and associated diseases. Emerging evidence demonstrates that innate immune cells, particularly ILCs, can develop memory to pregnancy. In the following sections, we review the characteristics and distribution of ILCs in the uterine compartment, describe innate immune memory, and discuss the potential relevance of this process to reproduction.

## Characteristics, Functions, and Distribution of ILCs

**Figure 1:**
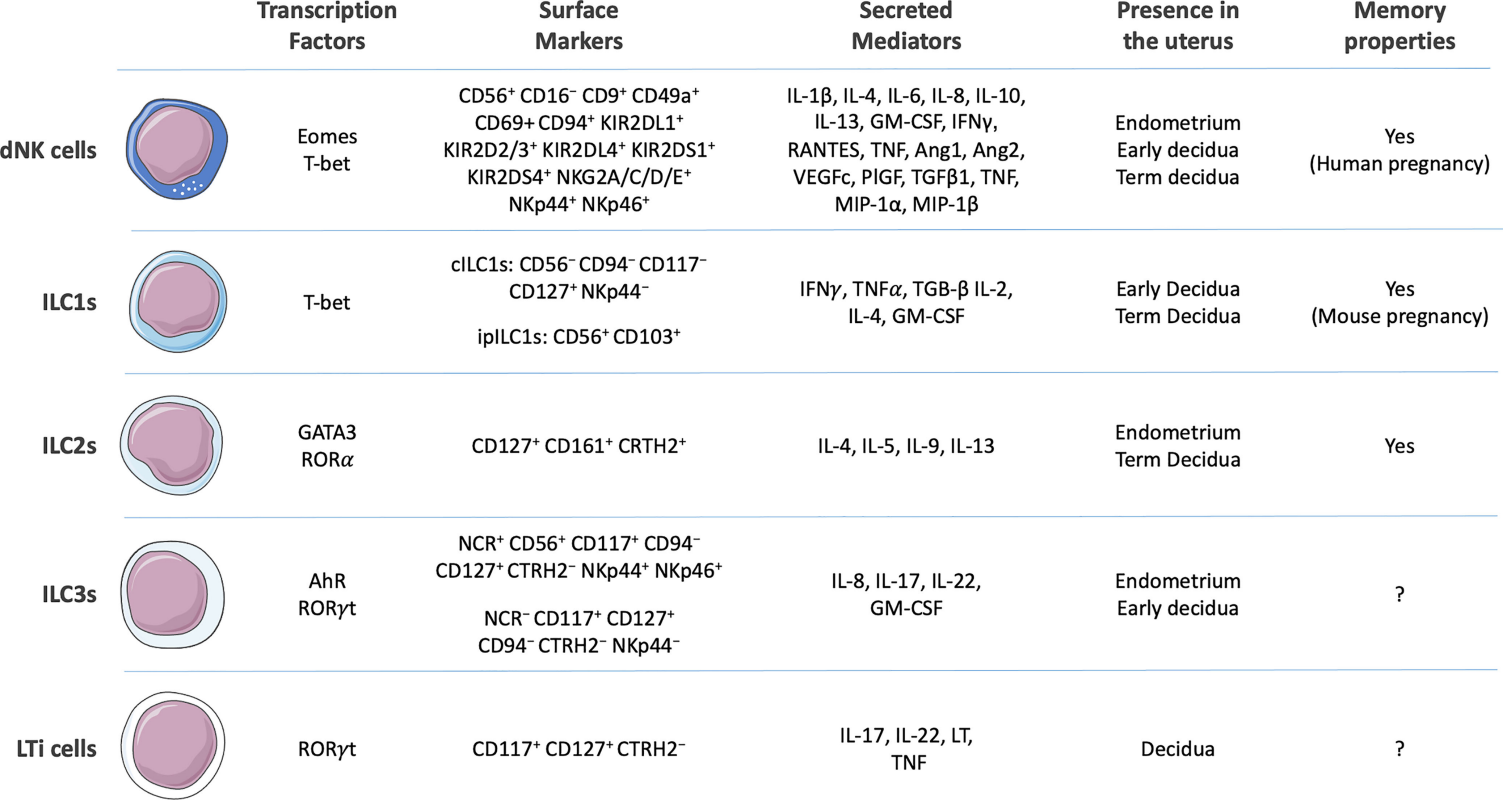
Surface markers, secreted cytokines, presence in the uterine environment, and capacity to develop memory of human decidual Natural Killer (dNK) cells (12–14) and generic human innate lymphoid cells (ILCs) 1 [conventional ILC1s (cILC1s) and intraepithelial (ipILC1s)], ILC2s, ILC3s, and lymphoid tissue inducer (LTi) cells (11, 15–17). Composed with images from: Smart Servier Medical Art (smart.servier.com).

Current studies are expanding our knowledge regarding ILC phenotypes, marker expression, transcriptional programs, and functional properties. Based on developmental transcription factor requirements and cytokine output, ILCs can be classified into five main groups: NK cells, ILC1s, ILC2s, ILC3s, and lymphoid tissue inducer (LTi) cells (**Figure 1** **)** (18, 19). Recently, regulatory ILCs (ILCregs) were described. However, it is not clear whether they represent a novel or a conventional subset that acquires immunoregulatory roles under certain biological circumstances (19, 20).

NK and ILC precursors develop independently through the divergence of common lymphoid progenitors. ILCs are considered innate counterparts of adaptive T lymphocytes that lack recombination activating gene (RAG)-dependent antigen receptors. ILC1s, ILC2s, ILC3s, and ILCregs mirror the function and cytokine profile of Th1, Th2, Th17, and regulatory T cells, respectively. Similarly, NK cells are considered counterparts of cytotoxic CD8^+^ T lymphocytes. ILC1s and Th1 cells play a key role in immune responses to intracellular pathogens (mainly viruses) and tumors; ILC2s and Th2 cells fight against large extracellular parasites and respond to allergens; ILC3s and Th17 cells combat extracellular microbes such as bacteria and fungi. ILCs also contribute to tissue homeostasis and remodeling as well as metabolic regulation (16, 19, 21–23). Although ILCs can circulate (24), they are essentially tissue-resident cells found in mucosal layers continuously exposed to microorganisms and potentially harmful agents. ILC functions at the endometrial mucosa are remarkably challenging since they also mediate unique processes related to fetal tolerance and placental development.

## NK Cells

NK cells are the founding members of the ILC family. Generated in the bone marrow and extramedullary sites, including the liver and uterus (25, 26), NK cells can detect and eliminate tumorigenic and virus-infected cells. Target cells are lysed through the release of granules containing granzymes and perforin. Antibody-dependent cell-mediated cytotoxicity, which connects innate and adaptive immunity, also mediates NK cell killing. The expression of eomesodermin (Eomes) and T-bet drives the differentiation of NK cells generating two major populations traditionally distinguished based on their expression of surface markers and cytotoxic capacity. CD3^−^CD56^dim^CD16^+^ NK cells present high cytotoxicity, whereas CD3^−^CD56^bright^CD16^−^ NK cells display low cytotoxicity and strong immunomodulatory properties (27). To regulate their functions, NK cells integrate signals from several stochastically expressed activating and inhibitory surface receptors. Among activating receptors present in human NK cells are NKp30, NKp44, NKp46, NKG2D, DNAM-1 and Killer Ig-like Receptors (KIRs), or their orthologs (Ly49 receptors) in mice. Another set of KIRs can also trigger inhibitory responses (12, 14).

NK cells have acquired specialized functions in different organs. In the uterine environment, they do not only fight against pathogens but also deplete senescent decidual cells that accumulate along the menstrual cycle (28). During pregnancy, NK cells act as biosensors of embryo viability and modulate maternal tolerance, endometrial vascular remodeling, and trophoblast invasion (29–34). Generically denominated uterine NK cells can be classified into endometrial (eNK) and decidual NK (dNK) cells present in the non-pregnant endometrium and pregnant decidua, respectively. eNK and dNK cells have different transcriptomic profiles and functional features (35, 36), likely due to distinct conditions present in the uterine environment and maternal body before and during pregnancy.

In humans, CD56^+^ eNK cells are scattered all over the endometrium and dramatically augment from the proliferative to the secretory phase of the menstrual cycle reaching up to around 70% of endometrial leukocytes (37–39). Large amounts of dNK cells remain in the decidua until the third trimester of pregnancy when they start to decrease (40). In mice, dNK cells concentrate during pregnancy in the mesometrial decidua where the chorioallantoic placenta develops (41, 42). Using an NK cell reporter model, Sojka et al. investigated the dynamics of dNK cells in the mouse uterus during pregnancy. The authors propose that the accumulation of these cells during early pregnancy occurs due to the proliferation of tissue-resident NK cells. Later on, peripheral blood NK cells seem to migrate to the uterine compartment since they are non-proliferative, but increase in numbers, to assist in placentation (43).

Three main subsets of NK cells were described in the human decidua using single-cell sequencing, dNK1 cells (characterized by the expression of CD39, CYP26A1, B4GALNT1, and CSF1); dNK2 (ANXA1, ITGB2, XCL1); dNK3 cells (CD160, KLRB1, CD103, XCL1, CCL5). HLA-C molecules on trophoblast cells can be recognized by both activating KIR2DS1 and KIR2DS4 as well as inhibitory KIR2DL1, KIR2DL2, and KIR2DL3 receptors expressed by dNK1 cells. dNK3 cells in turn contain KLRB1 and TIGIT, which may bind to CLEC2D and PVR present in trophoblast cells, indicating the capacity of these cells to cooperate during pregnancy. Furthermore, molecules produced by dNK1 (SPINK2, CD39, and CD73), dNK2, and dNK3 (ANX1) have anti-inflammatory properties inhibiting immune activation at the maternal-fetal interface (44). Further characterization of dNK cell in the human decidua through the combination of 28 markers assessed by mass cytometry revealed more than 4,700 phenotypes in a single donor. The functional significance of this diversity has not been fully appreciated (12, 45).

Small numbers of CD16^+^ NK cells are also present in the human endometrium, and increased levels of these cells are associated with infertility (39, 46, 47), potentially generating an aversive environment to embryo development. Single-cell RNA-sequencing (scRNA-seq) also revealed lower concentrations of CD39^−^CD18^−^ decidual NK (dNK) cells and CSF1^+^CD59^+^KIR^+^ dNK cells in women with unexplained recurrent pregnancy loss (48, 49).

## ILC1s

ILC1s were initially identified in tonsils and gut mucosa (50). Similar to Th1 cells, interferon γ (IFNγ) constitutes the main cytokine produced by ILC1s. They do not express Th2- and Th17-associated cytokines, segregating them from ILC2s and ILC3s, respectively (16, 18, 51). Moreover, unlike ILC2s and ILC3s, ILC1s do not express MHC class II molecules and are incapable of directly presenting antigens (52). ILC1 development requires T-box transcription factor (T-bet) (53). Since this feature is shared with NK cells, a proposed classification system includes NK cells as a subgroup of ILC1s. However, while both ILC1s and NK cells are positive for T-bet, only NK cells express the transcription factor Eomes (14), indicating they constitute distinct cell types.

Until now, little is known about ILC1 functions. ILC1s present non- to moderate-cytotoxic capabilities and secrete a broad spectrum of cytokines besides IFNγ, including TNF, IL-2, IL-4, TGF-β and GM-CSF. They also express various cytokine receptors (IL-7R, IL-17RD, IL-21R, TGFBR), suggesting their importance in immune regulation (51, 53, 54). In co-culture experiments, TGF-β and MMP9 produced by ILC1s contribute to epithelial and extracellular matrix remodeling of gut organoids (55). Most findings regarding ILC1s derive from pathological conditions. ILC1-derived IFNγ stimulates mononuclear phagocytes to mount a response to eradicate intracellular infections and contributes to chronic inflammation of the lungs and intestine (50, 51, 56). In addition, TRAIL-mediated ILC1 antiviral response plays a role against cytomegalovirus (CMV) (57). These data suggest that ILCs may participate in the endometrial inflammation and defense against pathogens. In the tumor microenvironment, TGF-β drives the conversion of Eomes^+^CD49a^−^CD49b^+^ NK cells favoring tumor immunosurveillance to Eomes^int^CD49a^+^CD49b^−^ ILC1s that are unable to control tumor growth, a mechanism by which tumors evade the innate immune system (58). In a similar manner, the conversion between subsets of ILCs could modulate their properties at the maternal-fetal interface.

In humans, ILC1s were identified within the epithelium of the oral and intestinal mucosa, liver, and tonsils (54, 59). There are contrasting data about the presence of uterine Eomes^-^ ILC1s in the human uterus. While some studies report their presence (60, 61), others could not identify them (62). ILC1s, ILC3s, and LTi-like cells are more abundant in the early pregnant human decidua. At term, ILC1s represent a minor subset while ILC2s are more prevalent (61). Based on single-cell transcriptional profiling, three main subpopulations of NK cells and ILC1s were described in the mouse uterus.

Eomes^-^CD49a^+^ ILC1s dominate before puberty; Eomes^+^CD49a^+^ tissue-resident NK cells resemble human dNK cells and are most abundant during early pregnancy; conventional Eomes^+^CD49a^-^ cytotoxic NK cells predominate after the establishment of the chorioallantoic placenta at mid-pregnancy (15). Eomes^+^CD49a^+^ NK cells can further be subdivided based on CD49b expression. While a population of CD49b^+^ cells increases during mouse gestation, another composed of CD49b^-^ cells decreases (60). Their functional significance remains to be determined.

## ILC2s

ILC2s are characterized by the expression of cell surface markers CD127 and CD161, chemo-attractant receptor homologous molecules expressed on Th2 cells (CRTH2), ST-2, an IL-33 receptor subunit, and IL-17RB, an IL-25 receptor subunit (18, 63). ILC2s require IL-7 and specific transcription factors, such as GATA-binding protein 3 (GATA3) and retinoic acid receptor-related orphan receptor-α (RORα), for their differentiation and function (14, 18). ILC2s respond to cytokines (IL-4, IL-25, IL-33, TL1A, and SCF), inflammatory mediators, neuronal factors, and hormones (64, 65). The production of Th2 cytokines (IL-4, IL-5, IL-9, IL-13) and amphiregulin (AREG) constitutes ILC2 main effector mediators with a diverse range of functions (66).

ILC2s are commonly located in the uterus, skin, lungs, gastrointestinal tract, and at small concentrations in circulating blood. Populations of CD127^+^ILC2s were described in both mouse and human uteri. In mice, these cells are located in the myometrium and increase during pregnancy (67). They express the IL-33 receptor ST2 and estrogen receptors. Upon estrogen treatment, ILC2s accumulate in the uterus of ovariectomized mice (68). In humans, ILC2 reside in the non-pregnant endometrium and decidua (67). At term, ILC2s seem to be the most abundant ILC type at the maternal-fetal interface after dNK cells. They are localized in both decidua basalis and parietalis (61) and present at higher numbers in the third trimester compared to the first and, therefore, may be more important toward the end of pregnancy. ILC2s secrete tissue repair factors such as AREG and IL-13, which support the homeostasis of the maternal-fetal interface (11). AREG has a tissue repair function by controlling the proliferation and differentiation of epithelial cells and epithelial barrier integrity (69). By releasing IL-5, ILC2s can control eosinophil responses and may promote remodeling of the uterine mucosa (14, 70).

## ILC3s

ILC3s express the surface marker CD117 and the transcription factor retinoic-acid-receptor-related orphan nuclear receptor γ t (RORγt). These cells mirror the features of Th17 lymphocytes regarding marker expression and cytokine output. ILC3s and ILC2s express major histocompatibility complex class II (MHCII) and can process and present antigens MHCII^+^. Two main ILC3 subpopulations are distinguished in humans by the presence of the natural cytotoxic receptor (NCR) NKp44: NKp44^+^ and NKp44^–^ ILC3s. In mice, the ILC3 population is sub-divided into NKp46^–^ and NKp46^+^ cells. Each subtype produces different sets of cytokines. NKp44^–^ ILC3s secrete IL-17 and TNF-alpha, whereas NKp44^+^ ILC3s produce IL- 8, IL-22, and GM-CSF (16, 71, 72). NKp44^+^ ILC3s expressing the chemokine receptor 6 (CCR6) and its ligand CCL20 also produce IL-17A and IL-22 and promote the accumulation at the inflammatory site of these cells and attraction of others, such as memory CD4^+^ T cells, regulatory Th17 cells, B cells, and dendritic cells (DCs) (73, 74).

ILC3s are abundant in mucosal compartments where their main functions are the defense against pathogens and epithelial tissue homeostasis. These cells are in constant interaction with the gut microbiota and have a protective effect against pathogenic bacteria *via* IL-22 secretion (75). Since the uterine environment is exposed to microbiota, ILCs could play a role in the regulation of endometrial homeostasis and response to microorganisms. During pregnancy, ILC3s are engaged in the recruitment of peripheral neutrophils into the uteurs, embryo implantation, and induction of maternal immune tolerance (14, 76). ILC3s were detected in the uterus of both virgin and pregnant mice (77). Likewise, in the human uterus, a population of NKp44^+^ ILC3s is present in non-pregnant endometrium and first-trimester decidua (62). However, a major difference observed between mice and humans concerns the localization of ILC2s and ILC3s in the uterus. In mouse pregnancy, ILC2 and ILC3s are found in the myometrium and ILC3s in the mesometrial lymphoid aggregate of pregnancy (MLAp) embedded within the uterine wall (62). In contrast, ILC2s and ILC3s are present in both human endometrium and decidua (60). Since these cells are not present in the mouse decidua, their effects on trophoblast cells should be limited compared to other ILCs on this species.

Potentially pro-inflammatory functions of ILCs have been proposed based on their increased proportion in chronic inflammatory conditions during pathological pregnancies. Elevated numbers of decidual ILC2s and ILC3s in the decidua have been associated with spontaneous preterm labor (61). Increased levels of IL-17 were detected in women with unexplained infertility and pregnancies complicated by preeclampsia and gestational diabetes. This process is not due to Th17 cells and may originate from the accumulation of ILC3s in the decidua (78, 79). IL-17 stimulates endometrial stromal cells to secrete IL-8 (80), which promotes the proliferation and survival of endometrial cells and acts on the chemoattraction and activation of neutrophils (81, 82). However, when excessively elevated in endometrial stromal cells, IL-8 impairs their ability to decidualize, leading to a non-receptive endometrium (83).

## LTi Cells

LTi cells, ILC3, and Th17 share the expression of RORγt. In addition, ILC3s and LTi cells express aryl hydrocarbon receptor and secrete IL-17 and IL-22, which are characteristic of ILC3s. However, different from ILC3s that depend on the transcription factor promyelocytic leukemia zinc finger (Plzf) to differentiate, LTi cells do not. LTi cells have a distinctive transcriptome and express CCR6, CCR7, CXCR5, CXCR6, IL-7Ra, LTα1β2, and RANKL at the cell surface (84, 85). During embryogenesis, LTi cells are involved in the development of lymph nodes and Peyer’s patches. They support the survival of memory CD4^+^ T cells *via* OX40L and CD30L (86, 87). IL-12-stimulated NKp46^+^ LTi cells encourage leukocyte extravasation in tumors through elevated expression of vascular adhesion molecules (88). CCL21 secreted by melanoma tumors promotes a tolerant environment by recruiting LTi-like cells and other regulatory immune cells (89).

Populations of LTi-like cells were described in the human decidua (CD127^+^CD117^+^) as well as in pregnant and non-pregnant mouse uterus (60, 62). Decidual LTi-like cells produce IL-17, tumor necrosis factor (TNF), and interact closely with decidual cells, stimulating the expression of ICAM-1 and VCAM-1(60). Considering the immunomodulatory properties of LTis cells, one could expect their involvement in immunological functions at the maternal-fetal interface.

## ILCregs

An ILC subset expressing IL-10 termed ILCreg was identified in both human and murine gut. ILCregs express the transcriptional regulator ID2 (Inhibitor Of DNA Binding 2, HLH Protein), ID3, and SOX4. TGF-β signaling is essential for their survival and expansion. ILCregs contribute to the resolution of intestinal inflammation by suppressing the activation of ILC1s and ILC3s *via* the secretion of IL-10 (19). Populations of ILCregs were also described in kidney, tonsils, lymph nodes, and cancer (90–92). Whether they are present in the uterus remains to be demonstrated.

## Innate Immune Memory

The traditional concepts characterizing innate and adaptive responses have been recently challenged. Innate immune cells present features previously attributed do adaptive immune cells, including clonal-like expansion, contraction, development of memory, and recall responses (17, 93, 94). Compelling evidence shows that innate immune cells can develop long-term adaptations (lasting for months or even years) leading to enhanced or lowered responses after subsequent stimulation, a process defined as innate immune memory or trained immunity (95).

Innate immune memory can be induced by pathogenic agents, microbiota organisms, cytokines such as IL-1, IL-12, IL-15, and IL-18, and hyperglycemia (96–98). Initial proof of the existence of adaptive features and memory formation in innate immune cells came from studies demonstrating that specific subsets of CD94^+^NKG2C^+^ NK cells in humans and Ly49H^+^ NK cells in mice expanded in response to murine CMV (MCMV) infection. After subsequent viral challenge, memory Ly49H^+^ NK cells triggered a recall response protecting against infection (93, 99, 100). Furthermore, contact hypersensitivity to haptens, regarded as a T cell-dependent process, was generated and maintained for up to four weeks in mice devoid of T cells. In contrast, mice lacking both T and NK cells did not respond. Adoptive transfer of NK cells from sensitized donors promoted a robust response in recipient animals after stimulation with previously encountered haptens (101). Since then, a rising number of reports has demonstrated the existence of trained immunity in other ILCs as well as in monocytes/macrophages, neutrophils, and DCs (17, 102–104).

Trained immunity occurs in liver ILC1s upon infection with MCMV. Viral glycoprotein m12 induces the expression of IL-18 receptor and distinct transcriptional and epigenetic profiles on these cells. Upon re-infection, memory ILC1s respond with higher IFNγ secretion (105). Transdermal hapten application promotes the generation of long-lived IL-7Rα^+^ memory ILC1s in the liver, which migrate to the effector site and generate robust allergic skin reactions following subsequent hapten contact (106). Trained ILC2s were identified in mouse models of allergic lung responses. After being challenged by allergen or cytokines, these cells display a greater response by increasing proliferation and producing larger amounts of cytokines, such as IL-5 and IL-13, compared to their naïve counterparts (107). The capacity of ILC3s, ILCregs, and LTi cells to develop memory has not yet been described.

## Mechanisms Regulating Innate Immune Memory

The development of trained immunity involves extensive epigenetic, transcriptional, and metabolic rewiring (95, 108). The epigenetic mechanisms include remodeling of chromatin architecture, post-translational modification of histones, DNA methylation, and expression of non-coding RNAs. Together they regulate the expression of genes controlling immunological responses. Analysis of chromatin accessibility and transcriptional profiling of differentiating and memory Ly48H^+^ NK cells challenged with MCMV showed that these two stages have distinct chromatin accessibility states and gene signatures. Memory cells present a poised regulatory program allowing them to respond in a faster way. The comparison between MCMV-stimulated NK and CD8^+^ T cells revealed that these cells share similar epigenetic processes to acquire memory (109).

Expansion of NKG2C^hi^ NK cells promoted by human CMV (HCMV) leads to epigenetic remodeling of the conserved non-coding sequence (CNS) 1, an enhancer in the IFNG locus, increasing its accessibility to transcription factors and IFNγ expression (110). The development of NK cell memory also involves histone modifications (lysine-4 mono-methylation (H3K4me1)) in an IFNG enhancer to sustain IFNγ production. Pharmacological inhibition of histone methyltransferases erases this epigenetic feature and NK cell memory (111). H3K4me1 modifications remain in NK and macrophages after the loss of signaling, allowing a faster and greater transcriptional response in case of re-stimulation (111, 112). In addition, phosphorylation of ATF7 (Activating Transcription Factor 7) triggers a long-term decrease in repressive histone methylation (H3K9me2) and an elevated expression of ATF7 target genes lasting after the end of stimulation (102).

miRNAs have been implicated in the development of innate memory. Activation of NK cells by MCMV infection elevates the expression of miR-155, which in turn downregulates NOXA and suppressor of cytokine signaling 1 (SOCS1), molecules related to immune cell survival and functions. NK cells deficient in this miRNA present impaired effector and memory properties (113). The development of macrophage memory (tolerance) through continuous exposition to LPS upregulates miR-221 and miR-222 levels. These miRNAs target Brg1 (brahma-related gene 1) to promote the downregulation of a subset of inflammatory genes *via* chromatin remodeling factors SWI/SNF (switch/sucrose non-fermentable) and STAT (signal transducer and activator of transcription) (114) to promote tolerance.

Cellular metabolism partakes in trained immunity by mediating epigenetics and other cellular processes. Metabolites can influence epigenetic features, conversely, epigenetic mechanisms contribute to maintaining cellular metabolism. When cells first recognize external stimuli, signaling cascades lead to elevated metabolism. The resulting metabolites can function as signaling molecules, cofactors, and substrates to modulate chromatin-modifying enzymes and direct transcriptional processes (115, 116). For example, fumarate and acetyl CoA can modulate epigenetic enzymes, such as histone demethylase lysine-specific demethylase 5 (KDM5) and histone acetyltransferases. Consequently, changes in histone methylation and acetylation of genes important to metabolism and innate immunity occur, resulting in memory establishment (95). Innate immune cells exhibit an up-regulation of metabolic pathways to become primed to secondary exposure. Metabolic rewiring provides for the proper energy needs of the cell, which is supplied by ATP through glycolysis and oxidative phosphorylation.

NK cells regulate their metabolic program to perform effector functions and develop memory. NK cell expansion promoted by MCMV causes mitochondrial depolarization and accumulation of reactive oxygen species. Following, during the development of NK memory, BNIP3 (BCL2/adenovirus E1B 19-kDa interacting protein 3) and BNIP3L (BNIP3-like) mediate the removal of dysfunctional mitochondria and reactive oxygen species through mitophagy promoting cell survival. Autophagy induction *via* inhibition of mTOR or activation of AMPK in a process dependent on ATG3 (Autophagy Related 3) amplifies the formation of memory NK cells (117). Virus-induced memory NK cells present elevated expression of genes regulating the electron-transport chain and increased oxidative mitochondrial respiration, mitochondrial membrane potential, and reserve respiratory capacity. ARID5B (AT-rich interaction domain 5B), a chromatin-modifying protein stimulated *via* DNA hypomethylation in memory NK cells, acts as a central regulator of these processes (117).

## Innate Immune Memory and Reproduction

Innate immune memory may occur already at hematopoietic stem cell or progenitor level in the bone marrow (118–120). This concept helps to explain the maintenance of long-lasting memory in short-lived immune cells. The transmission of immune memory to the next generation of cells allows stable maintenance of this process. H3K4me1 marks established in the HSC lineage were still present in terminally differentiated myeloid cells after cycles of DNA replication and cell division (121). A population of CD34^+^ hematopoietic stem cells residing in the human endometrium is committed to the differentiation of NK cells, as observed *in vitro* upon stimulation with a blend of stem cell factor, FMS-like tyrosine kinase ligand, IL-7, IL-15, and IL-21 or co-cultivation with decidual cells (26). The capacity of endometrial CD34^+^ cells to develop memory has not been investigated yet. If demonstrated, there may be relevant implications to uterine biology and pregnancy.

Conditions experienced during intrauterine life have a major impact on the development of chronic diseases later in life (122, 123). In connection, multiparity, birth order, and interpregnancy interval are associated with pregnancy outcome as well as health in childhood and adulthood (124–127). Distinct biological factors have been proposed to explain these phenomena. In a comprehensive compilation of reports, Thiele and collaborators (128) calculated a 4.2% increment of birth weight in second pregnancies independent of longer pregnancy duration (128). The gender and order of sibling birth also influence birth weight. A potential effect from male-specific immunological components was speculated (129), suggesting the development of immune memory to pregnancy.

Several reproductive diseases, such as preeclampsia, preterm birth, and intrauterine growth restriction, have a higher incidence in first than in second pregnancies (130–132). It was estimated that first uncomplicated pregnancies reduce the risk of complications in subsequent pregnancies by 35-65%. Contrariwise, complicated first pregnancies raise the risk of similarly affected second pregnancies by 2.2–3.2-fold (128). Although diverse, the aforementioned reproductive diseases may have impaired trophoblast invasion, deficient spiral artery remodeling, and altered immunological features. These processes are intertwined since immune cells can modulate endometrial vascular remodeling and trophoblast invasion. In this way, immunological processes affecting placental development can impact fetal growth and the occurrence of pregnancy complications (133, 134).

Several lines of investigation have been trailed to uncover causes leading to distinct outcomes in succeeding pregnancies encompassing morphological adaptations in the uterus, differences in trophoblast behavior, and immunological responses. For instance, lasting adaptations in the uterus of multiparous women were reported, characterized by changes in the internal elastic lamina and the proportion of muscular and connective tissue in the wall of spiral arteries (135). In addition, enhanced invasion of endovascular trophoblast cells was observed in the decidua of multigravidae versus primigravidae, indicating improved placentation in the former (136).

Exposure to paternal antigens represents a relevant factor for the development of pregnancy complications. Long-term engagement of a couple reduces preeclampsia incidence, however, such protection is lost when partners are changed (137, 138). Short periods of sexual contact, the use of physical contraception, and *in vitro* fertilization with oocyte or sperm donors, which implies the absence of previous paternal antigen exposition, have the opposite effect (139–142). The positive influence of previous pregnancies on reduced cases of preeclampsia in subsequent pregnancies last for up to eight years (143). Persisting changes in uterine immune cells (i.e. on their memory properties) may be related to improved pregnancy outcomes in second pregnancies. We speculate that innate and adaptive immune responses may be involved since both are stimulated by placental cells and seem to be beneficial to pregnancy (144, 145).

**Figure 2:**
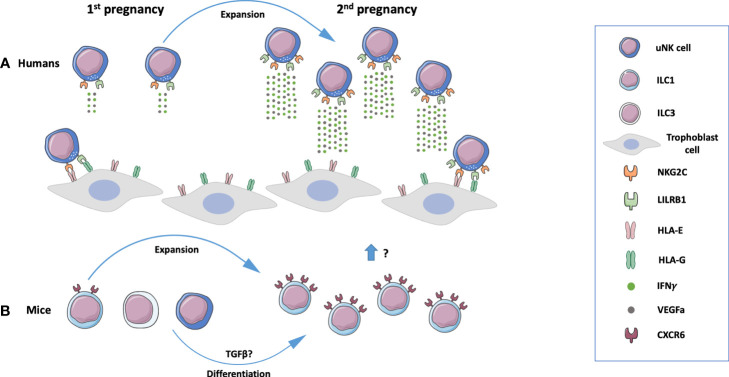
Memory properties of innate lymphoid cells (ILCs) in human and mouse pregnancy. **(A)** A population of NKG2C^hi^LILRB1^hi^ uterine Natural Killer (uNK) cells present in the human endometrium expands during subsequent pregnancies and produces high amounts of interferon y (IFNy) and VEGFa, mediators that contribute to placental development. These processes are mediated by NKG2C and LILRB1 on uNK cells that recognize respectively HLA-E and HLA-G molecules expressed by trophoblast cells (13). **(B)** In mice, a population of innate lymphoid cells 1 (ILC1s) expand during subsequent pregnancies. uNK cells and ILC3’s may be converted to ILC1s under the influence of TGFβ (15). Composed with images from: Smart Servier Medical Art (smart.servier.com).

Inceptive evidence concerning the establishment of innate immune memory on pregnancy was obtained in human dNK cells (**Figure 2**). The phenotype and functionality of dNK cells from first-trimester decidua of primigravid and multigravid women were compared. Through profiling of CXCR3, CXCR4, NKp44, NKp30, NKp46, NKG2D, NKG2C receptors by flow cytometry, a peculiar NKG2C^hi^ NK cell population was revealed and denominated pregnancy-trained decidual Natural Killer cells, which also express LILRB1 (Leukocyte Immunoglobulin Like Receptor B1) (13). CD94-NKG2C heterodimer binds to HLA-E molecules, which are expressed in trophoblast, endometrial, and endothelial cells (146, 147). LILRB1 recognizes HLA-G molecules on trophoblast cells at the maternal-fetal interface. Although this receptor has inhibitory functions on pbNK cells, it acts as an activator on dNK cells (148). NKG2C^hi^LILRB1^hi^ dNK cells present a distinct transcriptomic profile from NKG2C^low^ dNK cells. The engagement of NKG2C by HLA-E or LILRB1 by HLA-G promotes the secretion of high amounts of IFNγ and VEGFA in a process associated with epigenetic mechanisms. Conditioned medium from NKG2C^hi^LILRB1^hi^ dNK cells stimulates angiogenesis in both *in vitro* and *in vivo* models (13). IFNγ is a key cytokine required for reshaping decidual vasculature during placentation. Implantation sites in mice lacking IFNγ signaling fail to initiate pregnancy-induced modification of decidual arteries and display hypocellularity or decidual necrosis (29). The properties of PTdNK cells may contribute to explain improved placentation, higher birth weight, and reduced pregnancy complications in second pregnancies. A recent report provides data on the development of memory in ILC1s during mouse pregnancy (**Figure 2**). Eomes^–^CD49a^+^ ILC1s upregulate CXCR6, a receptor related to innate memory, and expand 4-5 times in second pregnancies (15). These cells have not been deeply characterized yet.

Although the acquisition of innate immune memory may have positive effects on pregnancy, one may speculate that impairments in the establishment of innate immune memory or the development of memory with inhibitory actions against pregnancy can be related to reproductive complications. Pregnancy induces long-term epigenetic memory in maternal T and NK cells characterized by differential methylation patterns of genes related to their differentiation and functions. These changes are impaired in preeclamptic women, indicating that alterations in innate and adaptive memory are associated with pregnancy diseases (149). The stimuli triggering innate immune memory in the uterine environment, their mechanisms, and repercussions to pregnancy warrant additional studies.

## Innate Immune Memory, Microorganisms, and Reproduction

So far, the implications of microbiota, pathogens, and vaccination on innate immune memory and its association with reproduction have been poorly interrogated. Chronic exposition of NK cells to both endogenous and exogenous ligands can modulate their memory properties (150). Such modulation is particularly relevant for the interaction with microbiota. NK cell and DC responses in germ-free mice are impaired when challenged with microbial components. Epigenetic mechanisms (histone acetylation and DNA methylation) were associated with defective expression of inflammatory markers, including type I interferons (151). These data demonstrate a contribution of microbiota to regulating immune responses. The female reproductive system contains a rich diversity of microorganisms, which has been correlated with reproductive health and disease (152). It may be expected that the uterine microbiota influences innate immune cells and their memory properties, consequently impacting fertility.

Pathogenic agents may also influence reproduction *via* innate immune cells and their memory properties. HCMV infection appears to be a priming factor in the development of pregnancy-trained NK cells. Comparisons between nulligravidae and multigravidae women that were HCMV-seronegative and -seropositive revealed increased percentages of LILRB1^+^NKG2C^+^ memory NK cells only in those previously infected by HCMV (153). Moreover, CXCR6 expressed in pregnancy memory ILC1s in mice is also associated with NK cell memory generated by viruses and haptens (15, 154). Further investigations may clarify if viral-induced innate immune memory has favorable or detrimental consequences for reproduction.

Infections and vaccinations can improve cellular responses to a second stimulus unrelated to the initial one. There is evidence that the BCG vaccine trains innate immune cells giving rise to immunity against cancer (155) and pathogens, including malaria, yellow fever, tuberculosis, and respiratory syncytial virus (156–159). A recent study in a mouse model showed that innate immune memory developed *via* the BCG vaccine previous to pregnancy impaired fetal growth. Reduced numbers of macrophages and NK cells were detected at the maternal-fetal interface together with decreased mRNA levels of CCR3 and LIF (160). This study provides evidence that pregnancy is influenced by immunological challenges experienced during life that develop innate memory responses.

## Concluding Remarks

Further studies on ILC biology as well as on the cellular and molecular mechanisms orchestrating innate immune memory and its implications to reproduction promise a fertile terrain of research. The potential existence of memory in other immune cells present in the uterine environment should also be explored. These data will contribute to clarifying the association of immune cells, microbiota, and pathogens with reproductive processes. Furthermore, the interaction of ILCs with trophoblast cells constitutes a central issue for pregnancy establishment, maternal immune tolerance, and placental development. Together, this knowledge may lead to novel therapeutic approaches addressing immune dysregulations that are relevant for infertility and pregnancy complications.

## Author Contributions

RF conceptualized the review’s subject. RF, RD-D, KP, and KU wrote the manuscript. RF and UM revised the final draft. All authors contributed to the article and approved the submitted version.

## Funding

The Placenta Lab has been supported by a grant from the German Research Society (DFG; grant number MA1550/12-1). RD-D is receiving a Master’s grant from the German Academic Exchange Service (DAAD).

## Conflict of Interest

The authors declare that the research was conducted in the absence of any commercial or financial relationships that could be construed as a potential conflict of interest.

## Publisher’s Note

All claims expressed in this article are solely those of the authors and do not necessarily represent those of their affiliated organizations, or those of the publisher, the editors and the reviewers. Any product that may be evaluated in this article, or claim that may be made by its manufacturer, is not guaranteed or endorsed by the publisher.
